# The standard versus prolonged dual antiplatelet therapy after the XINSORB bioresorbable scaffold implantation (SPARTA) trial: study protocol for a randomized controlled trial

**DOI:** 10.1186/s13063-022-07028-8

**Published:** 2023-01-20

**Authors:** Yizhe Wu, Jiasheng Yin, Chenguang Li, Wei Zhang, Li Shen, Lei Ge, Juying Qian, Junbo Ge

**Affiliations:** 1 Department of Cardiology, Zhongshan Hospital Fudan University, Shanghai Institute of Cardiovascular Diseases, 180 Fenglin Road, Shanghai, 200032 China; 2 National Clinical Research Center for Interventional Medicine, Shanghai, China; 3 Department of Biostatistics, School of Public Health, Fudan University, Shanghai, China

**Keywords:** Coronary artery disease, Percutaneous coronary intervention, Bioresorbable scaffold, Scaffold thrombosis

## Abstract

**Background:**

Dual antiplatelet therapy (DAPT) with aspirin and a P2Y12 inhibitor is the standard of care after coronary stenting, including coronary stenting involving bioresorbable scaffolds (BRSs). Current clinical guidelines recommend at least 12 months of DAPT after BRS implantation. However, the correlation between prolonged DAPT and net clinical benefits remains unknown.

**Methods:**

The SPARTA trial is designed to be a prospective, randomized, parallel-group, clinical trial. It aims to compare the benefits and risks of DAPT applied for either 12 or 36 months after XINSORB BRS implantation. The primary endpoints are the incidence of the composite endpoint of major adverse cardiac events (MACEs), including all-cause death, any myocardial infarction (MI), and all revascularizations, as well as Bleeding Academic Research Consortium Definition (BARC) type 3 or 5 bleeding events. The secondary endpoints of the study include the device-oriented composite endpoint of target lesion failure (defined as cardiac death, target vessel-related MI, or ischemia-driven target lesion revascularization), target vessel failure (defined as cardiac death, MI, or ischemia-driven target vessel revascularization), scaffold thrombosis, and minor bleeding events. This trial will enroll 2106 subjects treated with the XINSORB BRS only. All subjects will receive DAPT after the index procedure for 12 (± 1) months. Subjects without MACEs or major bleeding will be randomized to receive either 24 additional months of DAPT or aspirin alone.

**Discussion:**

This trial is designed to investigate the impact of extending the duration of DAPT up to 3 years after XINSORB BRS implantation by investigating the balance of risks and benefits in a broad population of treated patients.

**Trial registration:**

ClinicalTrials.gov NCT04501900. Registered on 6 August 2020.

**Supplementary Information:**

The online version contains supplementary material available at 10.1186/s13063-022-07028-8.

## Administrative information

Note: the numbers in curly brackets in this protocol refer to SPIRIT checklist item numbers. The order of the items has been modified to group similar items (see http://www.equator-network.org/reporting-guidelines/spirit-2013-statement-defining-standard-protocol-items-for-clinical-trials/).

**Table:** 

Title {1}	The standard versus prolonged dual antiplatelet therapy after the XINSORB bioresorbable scaffold implantation (SPARTA) trial: study protocol for a randomized controlled trial
Trial registration {2a and 2b}.	ClinicalTrial, NCT04501900. Registered 6 August 2020, https://clinicaltrials.gov/ct2/show/NCT04501900?term=04501900&recrs=ab&draw=2&rank=1
Protocol version {3}	V1.0, 1 Jun 2020
Funding {4}	Shanghai Clinical Research Center for Interventional Medicine (No. 19MC1910300)
Author details {5a}	Yizhe Wu^1,2^, Jiasheng Yin^1,2^, Chenguang Li^1,2^, Wei Zhang^3^, Lei Ge^1,2^, Li Shen^1,2^, Juying Qian^1,2^, and Junbo Ge^1,2^ ^1^ Shanghai Institute of Cardiovascular Diseases, Department of Cardiology, Zhongshan Hospital, Fudan University, Shanghai, China; ^2^ National Clinical Research Center for Interventional Medicine, Shanghai, China; 3 Department of Biostatistics, School of Public Health, Fudan University, Shanghai, China;
Name and contact information for the trial sponsor {5b}	Zhongshan Hospital Fudan University.
Role of sponsor {5c}	The sponsor—Zhongshan Hospital Fudan University—played part in study design; collection, management, analysis, and interpretation of data; writing of the report; and the decision to submit the report for publication

## Introduction

### Background and rationale {6a}

Bioresorbable scaffolds (BRSs, referring mainly to the Abbott biovascular scaffold [BVS], Abbott Vascular, Santa Clara, CA, US) are associated with increased risks of target lesion failure (TLF) and scaffold thrombosis according to recently published papers [1–3]. However, additional events of TLF and scaffold thrombosis related to BVSs were comparable to those observed with metallic Xience stents (Abbott Vascular, Santa Clara, CA, US) beyond the 3-year follow-up [4]. It was hypothesized that inflammation triggered by metabolites of poly-L-lactic acid (PLLA) would not terminate until full absorption of the struts. As a result, prolonged dual antiplatelet therapy (DAPT) was recommended after BRS implantation [5]. The XINSORB bioresorbable sirolimus-eluting scaffold (HuaAn Biotech, Shandong, China) is a contemporary BRS. It was approved by the China Food and Drug Administration (CFDA) in March 2020 because of its favorable long-term clinical outcomes [6–10]. More than 50% of XINSORB BRS-treated patients were still on DAPT at the 3-year follow-up. It was believed that prolonged DAPT played a critical role in maintaining low rates of TLF and scaffold thrombosis associated with the XINSORB BRS.

The results from the DAPT study showed that when compared with aspirin therapy alone, DAPT beyond 1 year after placement of a drug-eluting stent (DES) significantly reduced the risks of stent thrombosis and major adverse cardiovascular and cerebrovascular events [11]. However, prolonged DAPT did not show additional benefits in ischemia events compared with standard DAPT in stable coronary disease after DES deployment [11–14]. Furthermore, extending therapy beyond 1 year conferred an increased risk of bleeding. Any incremental benefit with regard to a reduction in the risk of clinical ischemia events and late stent thrombosis must therefore be weighed against an increased risk of bleeding. The use of DAPT in the DES era would not affect BRSs easily or identically. Hence, the correlation between ischemia and bleeding after BRS implantation remains unknown.

Given the lack of adequate randomized trial data, there is considerable uncertainty in the duration of DAPT after BRS implantation. The aim of the SPARTA trial is to investigate the impact of extending DAPT beyond 1 year after XINSORB BRS implantation by investigating the balance of risks and benefits in a broad population of treated patients.

### Trial design {8}

The SPARTA trial is designed to be a prospective, randomized, parallel-group, clinical trial. We will test the hypothesis that 36 months is superior to 12 months of DAPT.

### Objectives {7}

The primary objective of the SPARTA trial (NCT04501900) is to compare the effectiveness of 36 (prolonged) versus 12 (standard) months of DAPT after XINSORB BRS implantation. The safety objective is to compare the risk of Bleeding Academic Research Consortium Definition (BARC) type 3 or 5 bleeding in patients treated with 36 versus 12 months of DAPT [15].

## Methods

### Study settings {9}

The study is a multicenter clinical study carried out at approximately 30 sites in China. All sites will be academic hospitals (Refer to supplementary materials).

### Eligibility criteria {10}

**Fig. 1:**
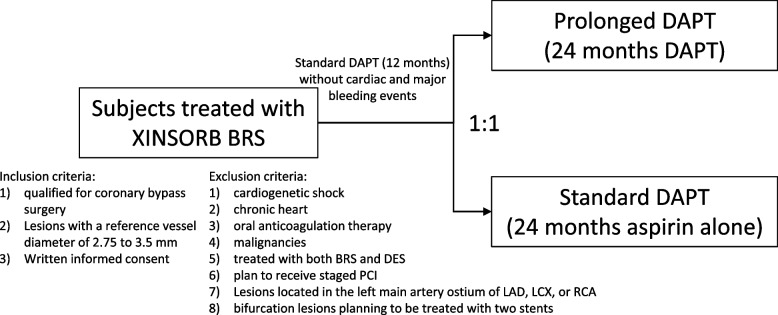
Consort flow diagram

Study subjects diagnosed with stable, unstable ischemic coronary disease or myocardial infarction (MI) planning to undergo percutaneous coronary intervention (PCI), and no contradiction to prolonged DAPT are eligible for this trial. All subjects will provide written informed consent to participate. Subjects will be enrolled in the study after 12 months (± 1 month) of the DAPT post index procedure. Subjects will be randomized to either discontinue a P2Y12 inhibitor (clopidogrel or ticagrelor) (12 months total) or receive a P2Y12 inhibitor for an additional 24 months (36 months total). Aspirin will be maintained throughout the entire study and can be replaced by cilostazol or indobufen if subjects are intolerant. The dosage of antiplatelet drugs will be determined according to the local standard of practice. Subjects will be treated with the XINSORB BRS only. Figure 1 shows the consort flow diagram of the study.

The major exclusion criteria included (1) cardiogenetic shock; (2) chronic heart failure with a left ventricular ejection fraction less than 30%; (3) subjects on warfarin or similar oral anticoagulation therapy; (4) known allergy or intolerance to aspirin, any P2Y12 inhibitor, heparin, contrast media, sirolimus, PLLA, or other allergens; (5) malignancies and other comorbid conditions with a life expectancy less than 5 years; (6) subjects treated with both BRS and DES; and (7) subjects who planned to receive staged PCI. Given the special properties and sizes of the BRSs, lesions with a reference vessel diameter of 2.75 to 3.5 mm will be included. Lesions located in the left main artery or less than 3 mm to the ostium of the left anterior descending (LAD) artery, the left circumflex (LCX) artery, or the right coronary artery (RCA) and bifurcation lesions (Medina 1,1,1) planning to be treated with two stents strategy will be excluded. The inclusion and exclusion criteria are detailed in Table 1.

**Table 1:** Inclusion and exclusion criteria

Inclusion criteria	(1). Subjects who received the XINSORB BRS and were then treated with DAPT for 12 months
	(2). Written informed consent obtained from the subjects
	(3). Candidates qualified for coronary bypass surgery
	(4). Lesions with a reference vessel diameter of 2.75 to 3.5 mm
Exclusion criteria	(1). Age ≤ 18 years
	(2). Cardiogenetic shock
	(3). Chronic heart failure with a left ventricular ejection fraction ≤ 30%
	(4). Oral anticoagulation therapy
	(5). Known allergy or intolerance to the study medications
	(6). Malignancies and other comorbid conditions with a life expectancy less than 5 years
	(7). Subjects treated with both BRS and DES during the index procedure
	(8). Pregnant women
	(9). Planned to receive staged PCI
	(10). Contemporaneous enrollment in a different clinical trial
	(11). Any revascularization within 1 year
	(12). Planned surgery necessitating the discontinuation of antiplatelet therapy within 36 months after enrollment
	(13). Unprotected left main artery
	(14). Lesions located at the ostium of the main coronary artery
	(15). Bifurcation lesions (Medina 1,1,1) planning to be treated with the two stents strategy

A total of 2106 subjects will be enrolled and randomized at approximately 30 centers in China in this manufacturer-run postmarketing study.

### Who will obtain informed consent? {26a}

Authorized physicians will obtain informed consent from the subjects before randomization.

### Additional consent provisions for the collection and use of participant data and biological specimens {26b}

The study will collect electrocardiography data and biological specimens, such as blood and urine, for liver and renal function, routine blood, blood cholesterol, and cardiac biomarker analysis, with the written consent of study subjects.

### Explanation for the choice of comparators {6b}

The results from the DAPT study showed that when compared with aspirin therapy alone, DAPT beyond 1 year after placement of a DES significantly reduced the risks of stent thrombosis and major adverse cardiovascular and cerebrovascular events. However, extending therapy beyond 1 year conferred an increased risk of bleeding. However, the use of DAPT in the DES era would not affect BRSs easily or identically. Hence, the correlation between ischemia and bleeding after BRS implantation remains unknown. Given the lack of adequate randomized trial data, there is considerable uncertainty in the duration of DAPT after BRS implantation. The aim of the SPARTA trial is to investigate the impact of extending DAPT beyond 1 year after XINSORB BRS implantation by investigating the balance of risks and benefits in a broad population of treated patients.

#### Scaffolds

As previously reported, the XINSORB BRS is composed of PLLA as its backbone. Poly-D-L-lactic acid (PDLLA) mixed with PLLA carrying sirolimus is coated on its struts. The dose of sirolimus administered was 8–16 μg/mm, depending on the length of the BRS. The thickness of the strut is 160 μm. The sizes of the XINSORB BRSs that will be used in this study are 2.75, 3.0, and 3.5 mm in diameter and 12, 15, 18, 23, and 28 mm in length.

### Intervention description {11a}

All subjects will take a loading dose of 300 mg clopidogrel or 180 mg ticagrelor plus 300 mg aspirin within 24 h before intervention. After the index procedure, a daily dose of a P2Y12 inhibitor (75 mg clopidogrel per day or 180 mg ticagrelor twice a day) as well as 100 mg aspirin per day will be administered over 12 months after the index procedure (observational period). Aspirin can be replaced by cilostazol (50 mg twice a day) or indobufen (100 mg twice a day) if subjects have a history of a gastric ulcer or bleeding. For the subjects who are eligible for randomization at 12 months (± 1 month), the prerandomization daily dose of the P2Y12 inhibitor will be continued or discontinued based on the randomization arm. Aspirin will be maintained for the duration of treatment. Subjects who switch from one type of P2Y12 inhibitor to another are eligible for enrollment if they have not changed within 6 months before randomization. All drugs will be prescribed to subjects routinely at the outpatient department of each center. Regular records of drug usage will be made throughout the entire study by investigators. Compliance will be reviewed by phone calls to subjects every 3 months from physicians to reconfirm the daily dose of each drug. Prasugrel has not yet been approved for the treatment of coronary artery disease in China.

All XINSORB BRS-treated subjects who are receiving 12 months (± 1 month) of the DAPT post index procedure and who are event-free (from death, MI, stroke, repeat revascularization, scaffold thrombosis, and BARC type 3 or 5 bleeding events) and who are compliant with DAPT (defined as no interruption more than 14 days) are eligible for randomization. The subjects will be randomized at a 1:1 ratio to receive either aspirin alone or a continuation of DAPT for an additional 24 months.

### Criteria for discontinuing or modifying allocated interventions {11b}

There will be no special criteria for discontinuing or modifying allocated interventions.

### Strategies to improve adherence to interventions {11c}

Subjects will be regularly followed up every 3 months after randomization by phone call or office visit to improve adherence. The drug table will be checked on site or by photos sent from subjects through message service. Blood tests and/or electrocardiograms will be performed every 3 to 6 months to monitor any abnormities.

### Relevant concomitant care permitted or prohibited during the trial {11d}

Subjects with ischemic symptoms will be admitted to the hospital. Repeat interventions were performed if lesions with restenosis were revealed. DAPT will be stopped if any BARC type 3 or 5 bleeding events occur and relevant care will be provided.

#### Follow-up

All randomized subjects will be followed up for 24 months after randomization. The subjects who are randomized to the prolonged DAPT arm will receive a daily dose of a P2Y12 inhibitor and aspirin for a total of 3 years (± 1 month). The other subjects will receive aspirin alone. All subjects will be contacted at 3, 6, 9, 12, 15, 18, 21, 24, 27, 30, 33, and 36 months through a phone call or office visit. Demographic, clinical, and procedural information at the time of enrollment as well as subsequent clinical endpoints, serious adverse events, concomitant medications, and antiplatelet therapy compliance will be obtained. Data will be continuously collected until the end of the study. All endpoints and events that occur after randomization will be adjudicated by an independent clinical events committee who is blinded to the study.

### Provisions for post-trial care {30}

The study will provide post-trial care and insurance for those who suffer harm from trial participation.

### Outcomes {12}

The primary endpoint of this study is the incidence of the composite endpoint, including all-cause death, any MI, and all revascularizations (major adverse cardiac events, MACEs) 24 months after randomization. MIs will be classified and adjudicated according to the Academic Research Consortium (ARC) definitions [16].

The secondary endpoints of the study include the device-oriented composite endpoint (DoCE) of TLF (cardiac death, target vessel-related myocardial infarction [TV-MI], or ischemia-driven target lesion revascularization [ID-TLR]), target vessel failure (TVF; cardiac death, MI, or ischemia-driven target vessel revascularization [ID-TVR]), the individual component endpoints of these endpoints, and scaffold thrombosis. Scaffold thrombosis will be defined as acute (< 24 h), subacute (1 to 30 days), late (30 days to 1 year), and very late (beyond 1 year), and the level of evidence (definite or probable) will be based on the ARC definitions [16].

Bleeding events are the secondary endpoint. BARC type 3 or 5 bleeding events at major bleeding events will include fatal bleeding, intracranial hemorrhage, cardiac tamponade, any transfusion with overt bleeding, a reduction in hemoglobin greater than 3 g/dl, and bleeding requiring surgery (excluding dental, nasal, skin, and hemorrhoid surgeries).

### Participant timeline {13}

**Fig. 2:**
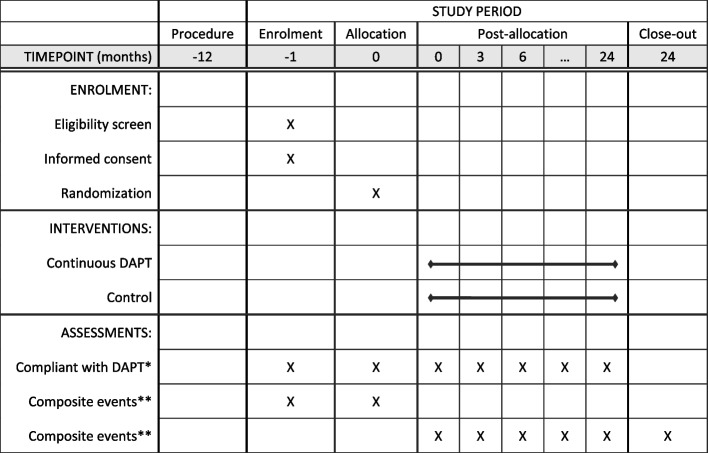
The schedule of enrolment, interventions, and assessments

The first enrollment is anticipated in the Autumn of 2022. It will take 2 years for all sites to complete enrollment. The time schedule of enrollment, interventions, assessments, and visits for subjects is shown in Fig. 2 below.

### Sample size {14}

Sample size was calculated by the primary endpoint of MACEs. The rates of MACEs related to the XINSORB BRS at the 1- and 3-year follow-ups were 2.5 and 4%, respectively, in the XINSORB RCT. The data were obtained from a randomized controlled study. The patients were relatively low risk. The lesions treated with the XINSORB BRS were simple to moderately complex. The rate of MACEs is definitely higher in the real-world population than in this patient population. However, the incidences of MACEs at 1 and 3 years in the ABSORB China study were 3.8 and 6.4%, respectively [17, 18]. The incidence of MACEs at 3 years in the ABSORB trials (ABSORB II, ABSORB Japan, ABSORB China, and ABSORB III) was 21.0% [3]. Assuming that the incidences of MACEs in the SPARTA trial will be 3.5% for the prolonged DAPT arm and 6.5% for the standard DAPT arm at 3 years, we will need a sample size of 649 subjects per arm to reveal a difference between the two arms by survival analysis based on a Cox model. We anticipate that 80% of subjects will be followed up for 36 months, and the randomization of 1624 subjects (812 per arm) will provide 80% power to demonstrate the superiority of prolonged (36 months) to standard (12 months) DAPT, with a 1-sided alpha value of 2.5%. Assuming that 12% of subjects who are initially enrolled will not be eligible for randomization at 12 months postprocedure because of any event or withdrawal, we need to enroll approximately 2106 subjects treated with XINSORB BRSs at the time of scaffold implantation.

### Recruitment {15}

Advertisement for recruitment will be posted on medias, like Wechat, Weibo, and Tiktok, so that any potential subjects will be reached. The recruitment of subjects was estimated to be completed in 2 years.

## Assignment of interventions: allocation

### Sequence generation {16a}

A computer-based Interactive Web Response System (IWRS) will be adopted. Randomization will be stratified by centers.

### Concealment mechanism {16b}

Authorized physicians followed corresponding treatments for enrolling subjects based on grouping information sent from IWRS.

### Implementation {16c}

Authorized physicians will generate the allocation sequence, enroll participants, and assign participants to interventions.

## Assignment of interventions: blinding

### Who will be blinded {17a}

Physicians and patients will not be blinded after assignment to interventions. However, all endpoints and events that occur after randomization will be adjudicated by an independent clinical events committee who is blinded to the study. Furthermore, data analysts will be blinded.

### Procedure for unblinding if needed {17b}

The design is open label, with only outcome assessors being blinded, so unblinding of the procedure will not occur.

## Data collection and management

### Plans for assessment and collection of outcomes {18a}

An electronic data capture (EDC) system will be used to collect study data. All baseline characteristics, clinical events, and outcomes will be reported to an independent committee. The committee members will be capable of assessing the information blindly. Subjects are mandatorily followed up regularly so necessary information can be acquired.

### Plans to promote participant retention and complete follow-up {18b}

Appropriate medical advice can be provided to subjects just in time through message or phone call if they adhere to the protocol and complete follow-up. To those who would be willing to take office visits, proper compensation will be provided. Subjects who suffer from any events or deviate from the protocol will be excluded from the study. However, they will still be monitored and cared for until the end of the study.

### Data management {19}

Both paper-based case report form (CRF) and EDC will be used in this study. CRF will be completed by authorized and trained physicians according to their medical history. It will be double checked by another physician relevant to this study. The paper-based CRF will be stored in the trial office securely. Then, the data will be entered into the database by authorized physicians for screening and randomization purposes.

### Confidentiality {27}

All information collected in this study will be strictly protected. Personal information about subjects including results of laboratory tests and procedural findings will be restricted in each center. Only those who is authorized by principle investigators can access the data. Subjects will be allocated an individual trial identification number and stored on a secure database to protect confidentiality before, during, and after the trial.

### Plans for collection, laboratory evaluation, and storage of biological specimens for genetic or molecular analysis in this trial/future use {33}

Biological specimens collected from subjects will be used only for laboratory testing and will be destroyed safely when the study ends. No genetic or molecular analysis is scheduled.

## Statistical analysis

### Statistical analysis of primary and secondary outcomes {20a}

All analyses will be performed on the intention-to-treat (ITT) population. The primary and secondary endpoints that are related to the time to an event will be assessed with Cox survival analysis. The primary analyses will compare the time to event from 12 to 36 months postprocedure between subjects randomized to 36 and 12 months DAPT using the stratified log-rank test. Kaplan‒Meier estimates of MACEs and other endpoints, as well as the 2-sided 95% confidence interval (CI) of the treatment difference in Kaplan‒Meier rates, will be presented for each treatment arm. Other secondary endpoints will be analyzed with the chi-square test or Fisher’s exact test for frequent comparisons. The two-sided significance level will be fixed at 5%. All tests will be performed with SAS version 9.4 (SAS Institute Inc., Cary, North Carolina, US).

### Interim analyses {21b}

There will be no planned interim analysis on MACEs in this study.

### Methods for additional analyses (e.g., subgroup analyses) {20b}

There will be no subgroup or adjusted analyses.

### Analysis methods to handle protocol nonadherence and missing data {20c}

Multiple imputation will be performed to handle missing data. Analysis will be performed based on the ITT population even if there might be crossovers.

### Plans to give access to the full protocol, participant-level data, and statistical code {31c}

The datasets analyzed during the current study and statistical code are available from the corresponding author on reasonable request, as is the full protocol.

## Oversight and monitoring

### Composition of the coordinating center and trial steering committee {5d}

The coordinating center and trial steering committee comprises at least 5 members, including senior physicians from the fields of cardiology and interventional cardiology, biostatisticians, and data keepers. There will be a chairman in the coordinating center and trial steering committee. He supervises the trial and will be responsible for all aspects of the local organization, including identifying potential recruits and obtaining consent. The members are not necessarily involved in the conduct of the trial. They will review the study (including reported serious adverse events) on a periodic basis. They will be unblinded to the treatment assignment but will receive results separately. The committee may stop the study for safety concerns at any time.

### Composition of the data monitoring committee, its role and reporting structure {21a}

A Data and Safety Monitoring Board (DSMB) will also contribute to the study. The DSMB is composed of at least 2 biostatisticians from the Department of Biostatistics, School of Public Health, Fudan University. The DSMB is independent from the sponsor and competing interests and will maintain the complete study database and perform all key analyses, including the primary efficacy and safety endpoints.

### Adverse event reporting and harms {22}

A clinical events committee (CEC) blinded to the assignment strategy will adjudicate all clinical events. The CEC will comprise physicians who are provided with all the data obtained from medical records necessary to perform optimal adjudications. Adverse events (AEs), serious adverse events (SAEs), and harms from interventions will be collected and reported to CEC by physicians. Furthermore, they will be reported to relevant regulatory bodies as needed, indicating expectedness, seriousness, severity, and causality.

### Frequency and plans for auditing trial conduct {23}

The ethics committee will meet every 6 months to communicate important protocol modifications to relevant parties if necessary. Members from joint meetings of the trial steering committee and DSMB will meet every month to audit trial conduct.

### Plans for communicating important protocol amendments to relevant parties (e.g., trial participants, ethical committees) {25}

Any changes to the protocol will be reported to the EC by the trial steering committee. Changes to the protocol, i.e., notifying sponsor and funder first, then the principal investigators (PI) will notify the centers and that a copy of the revised protocol will be sent to the PI to add to the investigator site file. Any deviations from the protocol will be fully documented using a breach report form. The protocol will also be updated in the clinical trial registry.

### Dissemination plans {31a}

The results of the study will be communicated to participants, healthcare professionals, the public, and other relevant groups via publications, reporting results in databases, data sharing arrangements, social media or through the sponsor.

## Discussion

It has been more than one decade since the first BRS was implanted. However, how long DAPT should be performed after BRS implantation remains unknown. It is clear that prolonged DAPT is associated with a low rate of ischemic events after DES implantation. However, inevitably increased bleeding caused by prolonged DAPT will offset the benefit from the treatment itself. It has been reported that BRSs are related to higher risks of TLF and scaffold thrombosis than metallic stents. The major cause of scaffold failure is MI. Hence, we hypothesized that prolonged DAPT would lower the risks of thrombosis and MI, subsequently decreasing ischemic events and improving clinical outcomes [19].

The earliest clinical trial of a BRS involved a minimum of only 6 months of DAPT after BVS implantation [20]. Fewer than 30% of patents were still on DAPT at the 4-year follow-up in the ABSORB II study [1]. MI and scaffold thrombosis occurred in 8.6 and 2.6% of BVS-treated patients, respectively. However, no late or very late scaffold thrombosis occurred in patients who never ceased DAPT. The benefit and need for prolonged DAPT after BRS implantation were then proposed [21]. In the XINSORB RCT, 59.0 and 54.1% of patients were still on DAPT at the 3- and 4-year follow-ups, respectively. The incidences of TLF, TV-MI, and scaffold thrombosis were significantly lower than those in the ABSORB serial trials. It was believed that prolonged DAPT was one of the reasons for maintaining favorable long-term clinical outcomes in patients who received the XINSORB BRS.

In contrast, prolonged DAPT will increase the incidence of bleeding while decreasing ischemic events, which may reduce the net clinical benefit of the treatment. However, no data regarding bleeding were collected from previous clinical trials of BRSs. The results obtained from metallic stents showed that extending the duration of DAPT was associated with an overall significantly higher risk of major bleeding than standard or short DAPT [22]. However, in the OPTIDUAL trial, the rates of major bleeding were low and very similar in both groups. Furthermore, mortality was not increased with an extended duration of DAPT [11]. As a result, the net clinical benefit of prolonged DAPT after DES implantation is still debated, and its net clinical benefit after BRS implantation remains unknown. Although the duration of DAPT tends to be short, such as 3 months or even 1 month, in selected patients treated with a new-generation DES and a new P2Y12 inhibitor [23–25], it is confusing and impossible to extrapolate experiences obtained from DESs to those of BRSs because of the different properties between these two devices.

It has been reported that the period of excess risks related to BRSs ceases at 3 years [4]. Before 3 years, adverse event rates associated with BVSs are clearly higher than those associated with Xience stents, many of which have been attributed to the increased risk of scaffold thrombosis. However, after 3 years, the clinical outcomes slightly favored BRSs, although the difference was not significant. Three years is the point after which organized struts are no longer detectable, which indicates full absorption of PLLA. The duration of DAPT should at least cover the BRS bioabsorption process to reduce the risks of MI and scaffold thrombosis.

Currently, BVS are no longer recommended in PCI. BRS raises concerns about late cardiovascular events and thrombosis compared with everolimus-eluting stents (EESs) in ABSORB II and III studies. Even optimal implantation techniques did not reduce the rates of death, MI, and revascularization. Abbott BVS withdrew from the global market in 2017 for catastrophic 3- and 4-year clinical outcomes. However, the differences in composite events between BRS and EES seemed to be narrowed beyond 3 years after the index procedure. Although careful selection of patients and optimal techniques of implantation may lead to promising results, prolonged DAPT still contributes to lower long-term adverse events. Last, a novel designed BRS, such as a rapidly biodegradable, thinner strut BRS, may overcome the limitations of currently used BRSs. We are looking forward to the performance of the next generation of BRS. The XINSORB BRS is a contemporary scaffold with Abbott BVS. The randomized control clinical trial of the XINSORB scaffold started in October 2014. The primary endpoint of 1-year in-segment late luminal loss (LLL) of XINSORB was noninferior to that of a traditional metallic sirolimus-eluting stent (SES) (0.19 ± 0.32 mm vs. 0.31 ± 0.41 mm, *P*_non-inferior_ = 0.003). At the 3-year follow-up, there was no significant difference in clinical outcomes in the XINSORB and SES arms, including TLF (4.0% vs. 6.2%, *P* = 0.29), cardiac death (1.0% vs. 0%, *P* = NA), TV-MI (1.0% vs. 0%, *P* = NA), and ID-TLR (3.5% vs. 6.2%, *P* = 0.19). The rate of confirmed/probable device thrombosis in XINSORB-treated patients was 1.0%. The XINSORB BRS is a unique device in the Chinese market. We need to identify the optimal duration of DAPT after BRS implantation.

## Trial status

The study has not yet begun. Recruitment is anticipated in autumn 2022 and ends in summer 2024.

## Supplementary Information

**Additional file 1.**

## Data Availability

Any data required to support the protocol can be supplied on request.

## Abbreviations

DAPT: Dual antiplatelet therapy
BRS: Bioresorbable scaffold
MACEs: Major adverse cardiac events
MI: Myocardial infarction
BARC: Bleeding Academic Research Consortium
TLF: Target lesion failure
DES: Drug-eluting stent
ARC: Academic Research Consortium
DoCE: Device-oriented composite endpoint
TV-MI: Target vessel-related myocardial infarction
ID-TLR: Ischemia-driven target lesion revascularization
TVF: Target vessel failure
ID-TVR: Ischemia-driven target vessel revascularization
