# Supplementary material for: The standard versus prolonged dual antiplatelet therapy after the XINSORB bioresorbable scaffold implantation (SPARTA) trial: study protocol for a randomized controlled trial
Source: Trials. 2023 Jan 20;24:49. doi: 10.1186/s13063-022-07028-8 (PMC9854195; doi:10.1186/s13063-022-07028-8)
Supplement: Supplementary file 1 — Additional file 1. [file 13063_2022_7028_MOESM1_ESM.docx]

Hospitals involved in SPATAR study:

Zhongshan Hospital Fudan University (Shanghai)

Tenth People’s Hospital of Tongji University (Shanghai)

Xinhua Hospital Affiliated to Shanghai Jiaotong University School of Medicine (Shanghai)

Tongji Hospital of Tongji University (Shanghai)

Shanghai Chest Hospital (Shanghai)

The Second Affiliated Hospital of Zhejiang University School of Medicine (Hangzhou)

Sir Run Run Shaw Hospital of Zhejiang University School of Medicine (Hangzhou)

The First Affiliated Hospital of Xi’an Jiantong University (Xi’an)

301 Hospital (Beijing)

Fuwai Hospital, CAMS & PUMC (Beijing)

Beijing Anzhen Hospital, Capital Medical University (Beijing)

Peking University First Hospital (Beijing)

Beijing Chao-yang Hospital, Capital Medical University (Beijing)

The People’s Hospital of Liaoning Province (Shenyang)

The First Hospital of China Medical University (Shenyang)

Shandong Provincial Hospital Affiliated to Shandong First Medical University (Ji’nan)

Qilu Hospital of Shandong University (Ji’nan)

Guangdong Provincial People’s Hospital (Guangzhou)

The First Affiliated Hospital of Xiamen University (Xiamen)

First Affiliated Hospital of Kunming Medical University (Kunming)

The First Affiliated Hospital with Nanjing Medical University (Nanjing)

The 1st Affiliated Hospital of Harbin Medical University (Harbin)

The 2nd Affiliated Hospital of Harbin Medical University (Harbin)

West China Hospital Sichuan University (Sichuan)

China-Japan Union Hospital of Jilin University (Jilin)

The First Hospital of Lanzhou University (Lanzhou)
